# The First Human Clinical Trial on the Skin Depigmentation Efficacy of Glycinamide Hydrochloride

**DOI:** 10.3390/biomedicines8080257

**Published:** 2020-07-31

**Authors:** Yong Chool Boo, Da Jung Jo, Chang Min Oh, Shin Young Lee, Young Mi Kim

**Affiliations:** 1 Department of Molecular Medicine, School of Medicine, Kyungpook National University, Daegu 41944, Korea; ckdals9669@naver.com; 2 Brain Korea (BK) 21 Plus Kyungpook National University (KNU) Biomedical Convergence Program, Kyungpook National University, Daegu 41944, Korea; 3 Cell and Matrix Research Institute, Kyungpook National University, Daegu 41944, Korea; 4 Ruby Crown Co., Ltd., Suite 505, Korea Mediventure Center, 76 Dongnae-ro, Dong-gu, Daegu 41061, Korea; lovelyroes@naver.com (S.Y.L.); help@rubycrown.com (Y.M.K.); 5 Dermapro Skin Research Center, Dermapro Ltd., Seoul 06684, Korea; djminu@naver.com

**Keywords:** melanin, glycinamide hydrochloride, depigmenting, pigmentation, skin lightening, whitening, peptide

## Abstract

A previous study identified certain low molecular anti-melanogenic peptides that share a common sequence with α-melanocyte stimulating hormone (MSH) and end with a glycinamide moiety. Glycinamide itself also showed anti-melanogenic activity in cell-based assays, but neither glycine nor acetyl glycinamide were active, which indicated a special structure and activity relationship. The aim of this study was to examine the skin depigmentation efficacy of glycinamide hydrochloride in human subjects. The primary skin irritation potential of glycinamide hydrochloride was evaluated by patch testing in 30 human subjects. The skin depigmentation efficacy of glycinamide hydrochloride was evaluated in a double-blinded clinical test in 21 human subjects. The test product and a control product were applied to designated sites on the right or left side of the face twice daily for eight weeks. Skin color parameters, i.e., the melanin index, the L* value (representing skin lightness), a* value (redness), and b* value (yellowness) were measured using instruments. The individual topology angle (ITA^o^, representing skin color) was calculated from L* and b values. The degree of skin pigmentation was visually assessed by two testers. The primary skin irritation test showed that a solution containing glycinamide hydrochloride up to 10% did not induce any adverse skin responses. In the efficacy test, the test product significantly reduced the melanin index, and increased L* value and ITA^o^ after two weeks of application relative to the baseline value at the start of the test. It also significantly lowered the degree of pigmentation after 6 weeks of application, relative to the baseline value. Differences in the melanin index, L* value, ITA^o^ and the degree of pigmentation between the test and control groups became statistically significant after six weeks or eight weeks of application. No signs of skin irritation were observed during the efficacy test. The present study suggests that glycinamide hydrochloride has great potential to be used in the control of skin hyperpigmentation.

## 1. Introduction

Human skin color is mainly determined by the content and distribution of various pigment substances, such as melanin, hemoglobin, and carotenoids [1]. Genetic factors primarily determine skin color, but it is also strongly influenced by acquired factors. Melanin is produced through a process in which an amino acid called L-tyrosine is metabolized in a series of enzymatic reactions in melanosomes, which are organelles of melanocytes. There are different types of melanin, such as eumelanin and pheomelanin, which impart different colors [2]. Melanosomes supply melanin to surrounding keratinocytes and, as a result, melanin is distributed throughout the skin and expressed to produce various skin color [3]. In addition to its effects on visual appearance of the skin, melanin plays an important role in protecting the body from the toxicity of ultraviolet (UV) rays [4]. Therefore, the metabolism of melanin in skin has become an important research topic from both a physiological and aesthetic perspective [5,6].

Abnormal melanin metabolism can cause various types of skin pigment diseases, which are divided into hyperpigmentation and hypopigmentation [7,8]. Hyperpigmentation occurs when melanin is excessively or unevenly accumulated due to inflammation, aging, UV rays, physical damage, and other internal/external stimulatory factors [9,10]. Meanwhile, genetic or epigenetic defects in melanin production can result in hypopigmentation, such as in albinism or vitiligo [11,12].

Prevention and treatment strategies for hyperpigmentation include photoprotection, pharmacotherapy, surgical treatment (chemical peeling and laser treatment), and cosmetic camouflage [13,14,15]. Chemical peeling and laser treatment are frequently performed, but they have side effects, such as dermatitis and recurring pigmentation [16,17]. Hydroquinone is primarily used as a pharmacotherapeutic, but it can potentially cause skin irritation, allergies, mutations, and cancer [18]. In the cosmetics field, skin lightening functional cosmetics containing arbutin, niacinamide, and vitamin C derivatives dominate, but the satisfaction of consumers regarding their safety and skin lightening efficacy is low [19]. This research team has searched for skin lightening agents from various natural sources and identified numerous natural compounds, such as p-coumaric acid, resveratrol, and luteolin 7-sulfate, which inhibit cellular melanin synthesis though various mechanisms [20,21,22,23].

Peptides are being increasingly used as active ingredients in dermatology and skincare products [24,25]. The smaller the peptide, the less expensive it is to manufacture, the higher its stability, and the more easily it is absorbed by the skin.

Our recent studies identified certain low molecular anti-melanogenic peptides [26,27]. In these studies, a special algorithm was used to predict the sequences of active peptides using a positional scanning synthetic peptide combination library [28,29]. Using this method, it was possible to identify anti-melanogenic peptides by evaluating the activity of 80 tetra-peptide pools instead of evaluating the activity of all 160,000 possible types of tetra-peptides [27]. The anti-melanogenic activity of the peptides was preliminarily evaluated in B16-F10 melanoma cells treated with α-melanocyte-stimulating hormone (MSH). The sequence of the active tetra-peptide was predicted to be R-(F/L)-(C/W)-(G/R)-NH_2_. Of the individual tetra-peptides tested, RFWG-NH_2_ and RLWG-NH_2_ showed high anti-melanogenic activity. The tetra-peptide FRWG-NH_2_, which has the same sequence as a part of α-MSH (acetyl-SYSMEHFRWGKPV-NH_2_), also showed similar activity. Among the tri-peptides tested, FWG-NH_2_, LWG-NH_2_, and RWG-NH_2_ were relatively active. The di-peptide WG-NH_2_ and the G-NH_2_ (glycinamide) retained their anti-melanogenic activity, while neither acety-G-NH_2_ nor G (glycine) was active. These low molecular anti-melanogenic peptides are thought to target the melanocortin 1 receptor (MC1R), because they have a sequence similar to that of α-MSH. These low molecular anti-melanogenic peptides may be very useful in the study of the MC1R-dependent physiological functions of melanocytes [30].

Glycinamide was shown to inhibit cellular melanin production very effectively by reducing the activation of cAMP-responsive element-binding protein (CREB) and the gene expression of microphthalmia-associated transcription factor (MITF), and tyrosinase (TYR) in response to α-MSH [27]. In the present study, we report the results of the first human skin application test of glycinamide (in the form of hydrochloride salt), which is the smallest of the low molecular anti-melanogenic peptides.

## 2. Materials and Methods

### 2.1. Materials

Glycinamide hydrochloride was purchased from Sigma–Aldrich (St. Louis, MO, USA), Hangzhou Dayangchem Co., Ltd. (Hangzhou, China), and Tokyo Chemical Industry Co., Ltd. (Tokyo, Japan). A test product (Melapep) containing 10% glycinamide hydrochloride as the active ingredient was provided by Ruby Crown. Co., Ltd. (www.rubycrown.com, Daegu, Korea). The control product comprised the same formulation without glycinamide hydrochloride.

### 2.2. High Performance Liquid Chromatography (HPLC)

Chromatographic analysis of the products was carried out using a Waters Alliance HPLC System (Waters, Milford, MA, USA) consisting of a Waters e2695 Separation Module and a Waters 2996 Photodiode Array Detector. The stationary phase was a 5 µm, 4.6 mm × 250 mm Hector-M C_18_ column (RS Tech Co., Daejeon, Korea), and the mobile phase was 20 mM phosphoric acid containing 5.0 mM sodium 1-octanesulfonate (pH 3.0). The flow rate of the mobile phase was 0.6 mL min^−1^. Detection wavelenth was 210 nm. A product was diluted 10 times with the mobile phase, and passed through a syringe filter (0.2 μm, cat#431219, Corning, Inc. NY, USA) prior to injection. The sample injection volume was 10 μL.

### 2.3. Primary Skin Irritation Test

A primary skin irritation test was conducted in accordance with personal care product council (PCPC) guidelines (2014) and standard operating procedures (SOP) of Dermapro Co., Ltd. (www.dermapro.co.kr, Seoul, Korea) to determine whether the test products irritated human skin. The test project (No. 1-220777-A-N-02-DICN19165) was approved on 1 August, 2019 by the bioethics committee of Dermapro Co., Ltd. and conducted in accordance with ethical principles based on the Helsinki declaration.

The subjects were selected from the healthy volunteers who meet the inclusion and exclusion criteria. We obtained the written informed consent from the subjects.

#### 2.3.1. Inclusion Criteria

(1) Healthy applicants aged 20−60 with no skin diseases. (2) Applicants who voluntarily signed a written consent form prior to the test after being informed of the purpose and contents of the test. (3) Applicants who were willing to cooperate with the requirements of the test and report immediately if there were any unexpected effects. (4) Applicants who could be followed up for the entire test period.

#### 2.3.2. Exclusion Criteria

(1) Women who were pregnant, nursing, or likely to become pregnant. (2) Applicants with a history of skin diseases, including atopic dermatitis, psoriasis, eczema, and seasonal allergies within the last month prior to the test. (3) Applicants with abnormal pigmentation, that may interfered with scoring at the test site. (4) Applicants taking anti-inflammatory, antihistamines, or immunosuppressive drugs. (5) Applicants who used topical anti-inflammatory drugs at the test site in the last two weeks before the test. (6) Applicants with skin diseases that may have interfered with the purpose of the test. (7) Applicants with a history of autoimmune diseases or immunodeficiency disorders. (8) Applicants who received immunotherapy in the last week prior to the test or were being planned to be treated during the test period. (9) Applicants who were receiving treatment for chronic diseases such as asthma and diabetes. (10) Applicants who were participating in other tests or who participated in a similar test for the last four weeks prior to the test. (11) Applicants who experienced skin irritation or allergies in response to attachment tape.

#### 2.3.3. Conditions of Participation

(1) The subjects were not allowed water on the test site while attaching the patch. (2) If the subject was required to take or use medicines, the test officer was to be notified. (3) Subject were to comply with the inspection schedule. (4) Subjects were to inform the tester of all abnormal skin symptoms that occurred during the test period.

#### 2.3.4. Criteria for Dropping Subjects during the Test Period

(1) If the subject voluntarily withdrew from the test due to a sudden accident, illness, pregnancy, etc. (2) If there was a serious adverse skin reaction caused by the test material. (3) If the subject did not follow the test protocol. (4) If the test could not be performed correctly due to special circumstances, e.g., if it was not possible to contact the subject.

#### 2.3.5. Patch Testing

The test materials (150 µL) were applied to Finn Chambers^®^ (Smart Practice, Hillerød, Denmark) using a Microman M250 pipette (Gilson, Villiers le bel, France). The chambers were attached to the back of the subjects and protected with Micropore tape (3M, St. Paul, MN, USA) for 24 h. The testers assessed skin reactions in the subjects two times, first 20 min. after patch removal and then 24 h later, using the skin reaction grading system described by Frosch and Kligman [31] with slight modification as follows: 0, no visible reaction; 1, slight spotty or diffuse erythema; 2, moderately uniform erythema; 3, intense erythema with edema; and, 4, intense erythema with edema and vesicles. The response score (R in %) was calculated as R = (Σ (observed reaction grade × frequency of response)/(total of four grades × total of two times observations × total number of subjects)) × 100 (%). The primary skin irritation potential of the test materials was described based on R, as follows: R < 0.87%, non-irritating/slight irritating; 0.87% ≤ R < 2.42%, mildly irritating; 2.42% ≤ R < 3.44%, moderately irritating; and, R ≥ 3.44%, severely irritating.

### 2.4. Skin Depigmentation Efficacy Test

A test was undertaken to evaluate the skin depigmentation efficacy of the test product. The test project (No. 1-220777-A-N-02-DICN19168) was approved on Aug 16, 2019 by the bioethics committee of Dermapro Co., Ltd. and conducted in accordance with ethical principles based on the Helsinki declaration. The test subjects were selected from healthy volunteers who meet the inclusion and exclusion criteria. We obtained the written informed consent from the patient (test subjects).

#### 2.4.1. Inclusion Criteria

(1) Men or women 20−60 years of age with hyperpigmentation, such as melasma or lentigo, on the face. (2) Healthy people with no acute or chronic physical diseases, including skin diseases. (3) Those who fell under type II, III, and IV of the Fitzpatrick skin type classification. (4) Applicants who were informed of the purpose, contents, etc. of the test, and voluntarily signed the test agreement. (5) Applicants who could be tracked during the test.

#### 2.4.2. Exclusion Criteria

(1) Those who were pregnant, nursing, or likely to become pregnant. (2) Those with a history of photoallergy or photosensitivity. (3) Those who used external preparations containing steroids for more than one month for the treatment of skin diseases. (4) Those who participated in similar tests within the 6 months prior to the test. (5) Those with sensitive and irritable skin. (6) Those with skin abnormalities such as spots, acne, erythema, capillary dilation, etc. at the test site. (7) Those who used the same or similar cosmetics or medicines at the test site within three months of the start of the test. (8) Those who consumed medicines or foods for skin lightening effects. (9) Those with atopic dermatitis or other infectious skin diseases. (10) Those who received medicinal treatment (skin peeling, Botox, other skin care, etc.) at the test site within 6 months prior to the test. (11) Those who received immunizations, antihistamines, anti-inflammatory agents, etc. within three months prior to the test. (12) Those who received systemic steroids, hormone therapy, or ray therapy within one month prior to the test. (13) People with chronic consumable diseases (asthma, diabetes, high blood pressure, etc.). (14) Those with jobs involving being exposed to sunlight for long periods of time. (15) Those who took drugs that induce photosensitivity. (16) Those who were judged to be unfit to participate in the test by the tester.

#### 2.4.3. Conditions of Participation

(1) Subjects were not to use cosmetics or medicines that claim functionality during the test period. (2) Subjects were to avoid exposure to more than normal levels of sunlight in daily life during the test period, and were to use sunscreen when going out. (3) Subjects were not to confuse the test products and were to apply them as prescribed. (4) Other than test products, subjects were not to change the cosmetic products that they used during the test period. (5) Subjects were to report all abnormal symptoms to the tester. (6) Subjects were to faithfully follow instructions regarding the use and restrictions of the products. (7) Subjects were to comply with the inspection schedule and confirm the use of the products to the tester.

#### 2.4.4. Criteria for Dropping Subjects during the Test Period

(1) If the subject voluntarily withdrew from the test due to a sudden accident, illness, pregnancy, etc. (2) If there was a serious adverse reaction caused by the test material. (3) If the subject did not comply with the test protocol. (4) If the test was hindered by special circumstances, e.g., if it was not possible to track the subject. (5) If the subject drank or smoked excessively, or was exposed to excessive UV.

#### 2.4.5. Application of Test Products

The test and control products (0.6 mL) were soaked into 4 × 3 cm^2^ of non-woven fabric sheets (product name, skin tissue type cotton) purchased from Blue and Pink (Gimpo-si, Korea), which were then individually packaged for use by the subjects.

The test was double-blinded; neither the testers nor the subjects were told which product was which. After receiving the test products in the same packaging from the provider, a test product manager labeled the packages with test codes in a separate room not accessible to the testers or the subjects. The testers received the test products from the test product manager in a blind state and the subjects also received the products from the tester in a blind state.

The block randomization method was used to allocate test products to the subjects. A block random allocation table (SSPP, SPSP, SPPS, PPSS, PSPS, and PSSP) was provided to a subject in order to determine the order of the test products, S or P, to be used by herself and three other subjects in the order.

The subjects used the test products after washing the face and before use of any other cosmetics twice a day, in the mornings and in the evenings, for eight weeks. The subjects attached the test and the control products to the designated sites on the left and right sides of the face, respectively (or vice versa), for 30 min., in accordance with the provider’s recommendation.

#### 2.4.6. Skin Color Assessments

The subjects visited the research center every two weeks for assessment of their skin. After washing their faces, the subjects rested for 20 min. before skin assessment in a laboratory maintained at 22 ± 2 °C and 50 ± 5% relative humidity.

The melanin index and erythema index were measured using a Mexameter^®^ MX18 (Courage + Khazaka electronic GmbH, Cologne, Germany). The mexameter probe emits three specific wavelengths of light (green, λ = 568 nm; red, λ = 660 nm; and infrared, λ = 880 nm), and a receiver measures the light reflected by the skin. The measurements were repeated three times and averaged.

Skin color is expressed using the Commission Internationale de l’Eclairage Lab color space based on the degree of lightness (L*), degree of green to red (a*), and degree of blue to yellow (b*) [32]. The skin color parameters, L*, a*, and b* were measured using a Spectrophotometer^®^ CM-2500d (Minolta, Tokyo, Japan) [32]. The individual typology angle (ITA°) representing skin color was calculated while using the equation: ITA° = (arc tangent [(L*−50)/b*])(180/3.14159) [33].

Visual assessment was conducted by two testers. They independently rated the degree of pigmentation of the skin on a scale from 0 to 9 (0, light; 9, dark; increments, 0.5). If the intra-class correlation coefficient value between two testers was greater than 0.8, the data were accepted and averaged values were used. Photographs of the skin were taken while using VISIA^®^ (Canfield Scientific, Inc., Fairfield, New Jersey, USA). For the evaluation of skin safety, the testers examined the skin for irritation and the subjects conducted a skin irritation self-assessment at each evaluation time point.

#### 2.4.7. Evaluation of Adverse Reactions of the Skin

During the eight-week trial period, the subjects visited the institute every two weeks. On each visit, the testers evaluated subjective symptoms by questioning whether the subjects had experienced adverse skin reactions, such as itching, prickling, tickling, burning, stinging, stiffness, and tightening over the past two weeks. The subjects were requested to immediately report if adverse skin reactions occurred between visit intervals. The testers also examined any adverse skin reactions, such as erythema, edema, size, and papules to assess objective symptoms.

### 2.5. Statistical Analysis

The data were analyzed using the SPSS software package (IBM, Chicago, IL, USA). Data are expressed as the Mean ± standard deviation (SD). The normal distribution of the data was verified using the Shapiro-Wilks test, and normality was accepted if Kurtosis and Skewness are within ± Pre-homogeneity of the test and control groups prior to the test was verified while using a paired t-test. The time-dependent changes compared to the baseline value, and the differences between the test and control groups at every time points were analyzed using the Repeated Measures ANOVA for parametric values, and Post-hoc Wilcoxon signed-rank test for non-parametric values. The change rate (%) from the baseline value was calculated, as follows; change rate = [value after treatment−baseline value before treatment)/baseline value before treatment] × 100 (%).

## 3. Results

### 3.1. Test Products

In this study, a test product containing 10% glycinamide hydrochloride and a control product of the same formulation without glycinamide hydrochloride were used. In addition to the main ingredient, the products contained glycerin, 1,2-hexanediol, and water. Table 1 shows the composition of the test product and control product.

**Figure 1:**
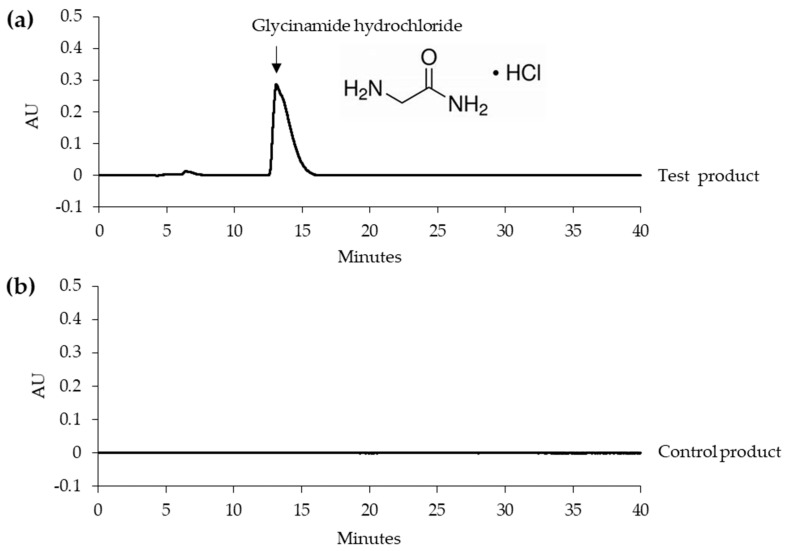
Typical HPLC chromatograms of the test and control products. (**a**) Test product. (**b**) Control product. The glycinamide hydrochloride peak is indicated by an arrow. The chemical structure of glycinamide hydrochloride is also shown.

Typical HPLC chromatograms of the test and control products are shown in Figure 1. The test product, but not the control product contained glycinamide hydrochloride. The glycinamide hydrochloride peak in the HPLC chromatogram is indicated by an arrow. Additionally, Figure 1 shows the chemical structure.

In HPLC, sodium 1-octanesulfonate was included in the mobile phase as an ion-pairing agent in order to enhance the retention of the protonated form of glycinamide, which was otherwise eluted very quickly from the reversed-phase octadecyl silica column. The presence of a UV absorbing amide bond in glycinamide hydrochloride allowed detection at 210 nm.

### 3.2. Primary Skin Irritation Test

In this test, 31 female subjects who fit the inclusion and exclusion criteria participated in the initial stages of the test; however, one subject dropped out (DSA-19046-10; voluntary withdrawal), so a total of 30 subjects completed the entire test. The average age of the subjects was 40.43 ± 7.13 years old; the oldest participant was 50 years old, and the youngest was 21 years old.

Occlusive patch testing was conducted for the purpose of evaluating the skin safety of the products. As shown in Table 2, subjects that were treated with the test, a control product, and a reference showed no adverse skin reactions at two different observation times. Response score in all cases was therefore zero, which indicated that the primary skin irritation potentials of these products are very low and they can be classified as “none-irritating/slightly irritating”.

### 3.3. Skin Depigmentation Efficacy Test

The test began with 23 female subjects, although two were subsequently eliminated (#16, voluntary withdrawal; #18, non-compliance with protocol), so the final number of participants was 21. Information on the subjects was gathered in a questionnaire-based survey. The average age of the subjects was 48.43 ± 4.14 years old; the oldest participant was 55 years old; and, the youngest was 41 years old. Table 3 shows the skin characteristics of the subjects surveyed.

A double-blinded test was conducted in order to evaluate the depigmentation efficacy of the test product. Neither the tester and nor the subject were able to distinguish between the test product and the control product, and the clinical trial was conducted according to the prescribed method. The subjects applied the test product and the control product to the left or right side of the face twice a day for eight weeks, and visited the institute every two weeks for skin assessment.

**Figure 2:**
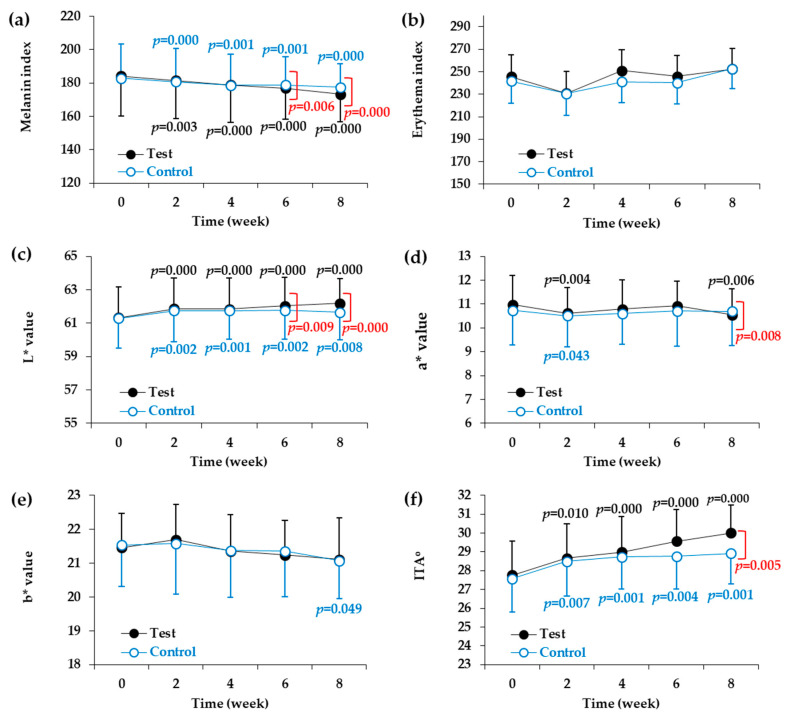
Changes in skin color parameters of the facial skin during application of test and control products. Melanin index (**a**), erythema index (**b**), L* value (light ness) (**c**), a* value (redness) (**d**), and b* value (yellowness) (**e**) were determined by instrumental analysis. ITA° (skin color) (**f**) was calculated from L* and b* values. Skin color parameters are plotted against time (weeks). Data are presented as mean ± SD (*n* = 21). The *p* values of the control group and test group versus the baseline values before the start of the test are shown in blue and black letters, respectively, if they are less than 0.The *p* values of the test group versus the control group at each time point are shown in red letters if they are less than 0.05.

Two types of devices were used for the instrumental evaluation of skin color parameters. First, the melanin index and the erythema index were measured using a mexameter. As shown in Table 4 and Figure 2a, significant reductions in melanin index were observed at two, four, six, and eight weeks in both the test and control groups compared to the baseline values before the start of the test. When the test product was applied for two, four, six, and eight weeks, there was a significant reduction in the melanin index compared to the baseline value. The melanin index reduction rates were 1.48% after two weeks, 2.87% after four weeks, 3.92% after six weeks, and 5.88% after eight weeks. Significant differences were found between the test and control groups at six and eight weeks after the start of the test. On the other hand, there were no changes in the erythema index in either the test group or the control group as compared to baseline values measured before the start of the test (Table 5, and Figure 2b).

We also used a spectrophotometer to measure L*, a*, and b* values, which represented skin lightness, redness, and yellowness, respectively. As shown in Table 6 and Figure 2c, significant increases in skin lightness (L* value) were observed at two, four, six, and eight weeks in both the test and control groups compared to the baseline values before the start of the test. Compared to the baseline values, the test product increased the L* value by 0.88% after two weeks, 0.85% after four weeks, 1.16% after six weeks, and 1.40% after eight weeks. Significant differences were found between the test product and control product at six and eight weeks after the start of the test. On the other hand, although there some changes in the a* and b* values in both the test group and the control group compared to before the test was started, these changes were not consistent (Table 7 and Table 8, Figure 2d,e). The a* value decreased temporarily in the second week in the control group and in the second and eighth weeks in the test group. The a* value of the test group was lower than that of the control group in the eighth week. The b* value decreased in the eighth week only in the control group, and no difference in b* value was observed between the control and test groups.

The ITA° calculated from the L* and b* values was used to express skin color. The higher the ITA°, the lighter is the skin color. As shown in Table 9 and Figure 2f, significant changes in skin color were observed at two weeks, four weeks, six weeks, and eight weeks in both the test group and the control group compared to the baseline values before the start of the test. When compared to the baseline values, the test product increased the ITA° by 3.28% after two weeks, 4.43% after four weeks, 6.48% after six weeks, and 8.07% after eight weeks. A significant difference in ITA° was also found between the test product and control products at eight weeks after the start of the test.

**Figure 3:**
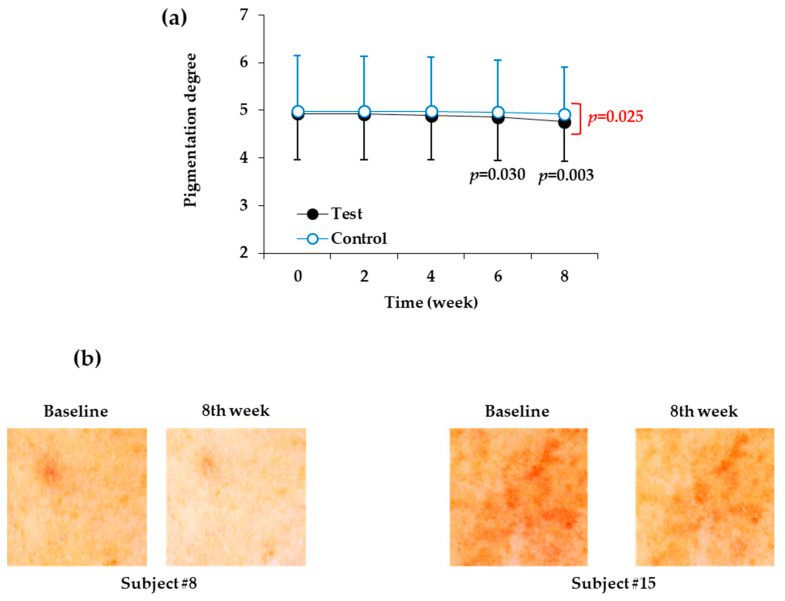
Changes in the visually assessed degree of pigmentation of the facial skin during application of the test and control products. (**a**) Degree of pigmentation was visually assessed by two independent testers and plotted against time (weeks). Data are presented as mean ± SD (*n* = 21). The *p* values of the test group versus the baseline value before the start of the test are shown in black letters if they are less than 0.The *p* values of the test group versus control group at each time point are shown in red letters if they are less than 0.05. (**b**) Images of the test skin sites to which the test or control products were applied for eight weeks. Representative skin images of human subjects # 8 and #15 before and after the treatment with test product for eight weeks are shown.

As shown in Table 10 and Figure 3a, the skin site at which the control product was applied did not significantly change in terms of degree of pigmentation for eight weeks, but the degree of pigmentation at the skin site at which the test product was applied decreased significantly after six weeks and eight weeks. When the test product was applied for six and eight weeks, the degree of pigmentation was reduced by 1.42% and 3.45%, respectively, relative to the baseline value before the start of the test. In the eighth week after the start of the test, there was also a significant difference in the degree of pigmentation between the test product and the control product. Figure 3b shows images of two representative human subjects.

There were no adverse reactions to the test and control products reported by the subjects or detected by the testers over the eight weeks.

## 4. Discussion

This study is the first to report the safety and depigmentation efficacy of glycinamide hydrochloride applied to human facial skin. In the primary skin irritation test, a test product containing 10% glycinamide hydrochloride did not induce any adverse reactions in 30 human subjects. In the double-blinded efficacy test, the test product and control product were used on the left and right sides, respectively, of the face in 21 subjects, randomly assigned to different groups. During the eight week-long test period, the skin sites at which the test product was applied became lighter than the sites at which the control product was applied. Both visual assessment of the degree of pigmentation, and instrumental evaluation of melanin index, skin lightness, and skin color supported the depigmentation efficacy of the test product containing glycinamide hydrochloride.

The human skin depigmentation efficacy of a product can be evaluated while using artificial tanning and natural hyperpigmentation models [34,35,36]. In the present study, using a natural pigmentation model, changes in skin color parameters in the test and control groups over the eight weeks of test were tracked at two week intervals. The control product, which did not contain glycinamide hydrochloride, also produced significant changes in melanin index, L* value, and ITA ^o^ from two weeks after the start of the test. The exact cause of this is currently unclear and several factors might have contributed to the changes. The ingredients contained in the control product, such as glycerin and 1,2-hexanediol, might have altered skin surface condition, improving cleanness, luster, and brightness. Nonetheless, additional changes in the parameters mentioned above after two weeks were not significant in the control group. In addition, the degree of pigmentation (evaluated visually) was not significantly reduced by the control product when compared to before the start of the test. Therefore, it is considered that the control product has a very weak skin lightening effect if any.

In the case of the test product, in addition to a significant change in the melanin index, L* value, and ITA^o^ at two weeks after the start of the test, the change tended to increase gradually thereafter. Furthermore, the degree of pigmentation reached a significantly lower level than the baseline value. This suggests that use of the test product can improve skin color when compared to use of no product.

From the sixth week of the start of the test, there was a significant difference in the melanin index and L* value between the test group and the control group and, at eight weeks, there was also a difference in ITA^o^ and the degree of pigmentation. These results support the conclusion that the difference in efficacy between the test and control products was due to the presence or absence of glycinamide hydrochloride; therefore, the skin depigmentation efficacy of the test product was due to its main component, glycinamide hydrochloride.

In this study, glycinamide hydrochloride solution soaked into non-woven fabric sheets was applied to the face, and positive results were obtained regarding its skin depigmentation efficacy. This application method turned out to be effective, but it is worth investigating whether changing the formulation and application method would improve the efficacy of the product. In addition to topical application onto skin, oral administration and injection methods need to be examined in order to determine their clinical efficacy in future studies.

Statistical analysis for the differences between the test and control groups was conducted using the Repeated Measures ANOVA with paired comparisons. Additionally, the inter-group differences of melanin index, L* value, ITA^o^, and pigmentation degree were all statistically significant after 8-week treatments. In the current study, the melanin index reduction rates by the test product containing 10% glycinamide hydrochloride was 5.88% (baseline, 184.03 ± 22.70; eighth week, 173.30 ± 20.79). Watanabe et al. reported skin-whitening effects of topical oxidized glutathione in a double-blind and placebo-controlled clinical trial in thirty healthy women [36]. Topical treatments of a test product containing 2% oxidized glutathione for 10 weeks reduced melanin index by 10.7% (baseline, 272.77 ± 26.17; 10th week, 243.47 ± 26.31) [36]. Even though the test conditions, such as baseline melanin index, test dose, and duration of test, are different each other, the indirect comparison of these two studies suggest that glycinamide hydrochloride has potential to be used as a hypopigmenting agent. Further studies are needed to optimize its dose, formulation, and application method to achieve better performance in skin depigmentation.

The steady state skin color reflects the relative ratio of the amount of melanin supplied from melanocytes to the amount of melanin lost due to desquamation of the skin’s keratin. Therefore, the inhibition of melanin synthesis or enhancement of melanin loss are two possible strategies for hyperpigmentation therapy. Glycinamide hydrochloride is a useful substance for the former strategy, because it can inhibit melanin synthesis by reducing the levels of TYR and other enzymes that are involved in melanin synthesis in melanocytes [27]. If so, combining glycinamide hydrochloride with skin peeling agents, such as glycolic acid [37,38], may result in further increases in depigmentation efficacy due to synergism between substances with different mechanisms of action.

Many existing depigmentation agents reduce the synthesis of melanin by inhibiting the intracellular signal transduction process responsible for TYR gene expression or inhibiting the activity of TYR directly [39,40]. However, glycinamide hydrochloride can prevent the binding of a hormone to its receptors and block the initiation of intracellular signal transduction processes, thereby inhibiting the expression of TYR [27]. Accordingly, glycinamide hydrochloride can act via a different mechanism from many existing depigmenting agents, and synergistic effects are expected if it is used in combination with other depigmenting agents.

Proopiomelanocortin-derived peptide hormones, such as α-MSH, β-MSH, and adrenocorticotrophic hormone (ACTH), regulate skin pigmentation, inflammation and fibrosis [8,30,41]. On the binding of these agonists to MC1R and the consecutive activations of adenylate cyclase (AC) and protein kinase A (PKA) phosphorylates CREB transcription factor, which, in turn, induces MITF expression [42]. MITF expression is also induced by other signaling pathways that involve Wnt/Frizzled/glycogen synthase kinase 3β/β-catenin cascade, and stem cell factor/c-Kit/mitogen-activated protein kinases cascade [43,44]. MITF directs not only gene expression of melanogenic enzymes, but also the biogenesis of melanosomes [2,45].

**Figure 4:**
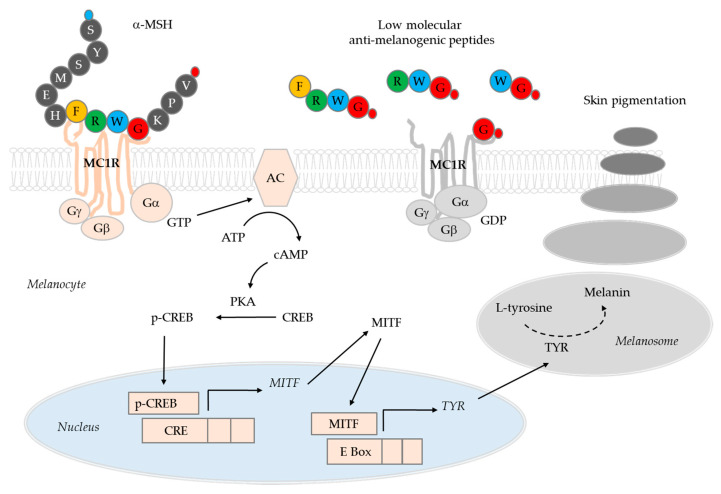
A working hypothesis for the mechanism of action of low molecular anti-melanogenic peptides. α-Melanocyte-stimulating hormone (MSH) binds to the G-protein coupled MC1R receptor to activate adenylate cyclase (AC). The resulting cAMP-dependent activation of protein kinase A (PKA) increases the phosphorylation of cAMP-responsive element-binding protein (CREB), and then p-CREB enters the nucleus to increase the expression of microphthalmia-associated transcription factor (MITF). MITF transcription factors increase melanin synthesis by inducing the expression of tyrosinase (TYR) which catalyzes the key reactions in melanin synthesis in melanosomes. Melanosomes migrate from melanocytes to epidermal keratinocytes, resulting in melanin deposition and skin pigmentation. In a previous study, we identified low molecular anti-melanogenic peptides, such as FRWG-NH_2_, RLWG-NH2, FRWG-NH2, FWG-NH2, LWG-NH2, RWG-NH_2_, WG-NH_2_, and G-NH_2_ (glycinamide), some of which share common sequences with α-MSH (acetyl-SYSMEHFRWGKPV-NH_2_) [27]. In the cartoons of α-MSH and anti-melanogenic peptides, amino acids are represented with single letter symbols in closed circles. The small blue and red circles represent an acetyl group and an amide group, respectively. In the present study, we reported that glycinamide (G-NH_2_) in its hydrochloride salt form exhibited significant human skin depigmentation efficacy. Further studies are needed in order to examine the depigmentation efficacy of other low molecular anti-melanogenic peptides.

The low molecular anti-melanogenic peptides identified in the previous study include FRWG-NH_2_, RWG-NH_2_, WG-NH_2_, and G-NH_2_ (glycinamide), which share common sequences with α-MSH (acetyl-SYSMEHFRWGKPV-NH_2_) [27]. It was hypothesized that these low molecular anti-melanogenic peptides might interfere with the receptor binding of α-MSH in a competitive manner thereby blocking the initiation of intracellular signal transduction processes (Figure 4). If the low molecular anti-melanogenic peptides act as MC1R antagonists, they may inhibit the activation of MC1R by other agonists, such as β-MSH and ACTH as well, and affect other physiological evets associate with inflammation and fibrosis as well as pigmentation [30].

Although more direct evidence is needed for the mechanism of action, the present study demonstrates that the hydrochloride salt form of glycinamide, the smallest essential moiety of the anti-melanogenic peptides, exhibited skin depigmentation efficacy without causing skin irritation in humans. Further studies are needed in order to examine the depigmentation efficacy of the other anti-melanogenic tetra-peptides, tri-peptides, and a di-peptide mentioned above.

In conclusion, the present study suggests that glycinamide hydrochloride has great potential to be used in the control of skin hyperpigmentation.

## Figures and Tables

**Table 1:** Composition of test and control products (%, *w/w*).

Ingredients	Test Product	Control Product
Glycinamide hydrochloride	10	-
Glycerin	10	10
1,2-Hexandiol	1.5	1.5
Water	77.5	87.5

**Table 2:** Results of primary human skin irritation test (*n* = 30).

Test Materials	Frequency of Skin Reaction								Response Score (R in %)
	24 h				48 h				
Reaction grade:	1	2	3	4	1	2	3	4	
Test product (Melapep)	0	0	0	0	0	0	0	0	0.0
Control product (base)	0	0	0	0	0	0	0	0	0.0
Negative control (squalane)	0	0	0	0	0	0	0	0	0.0

**Table 3:** Skin characteristics of subjects in the skin depigmentation efficacy test (*n* = 21).

Parameters	Criteria	Frequency (*n*)	Percentage (%)
Age	40 s	10	47.62
	50 s	11	62.38
Fitzpatrick skin type	II	1	4.76
	III	15	71.43
	IV	5	23.81
Pigmentation	Little	1	4.76
	Moderate	11	52.38
	Much	9	42.86
Skin type	Dry	10	47.62
	Moderate	8	38.10
	Oily	0	0
	Dry & oily	3	14.29
	Problematic	0	0
Irritation sensitivity	Yes	2	9.52
	No	19	90.48
Itching sensitivity	Yes	0	0
	No	21	100
Adverse reaction experience	Yes	0	0
	No	21	100
Skin moisture	Excessive	0	0
	Moderate	9	42.86
	Insufficient	12	57.14
Skin sebum	Excessive	1	4.76
	Moderate	16	76.19
	Insufficient	4	19.05
Skin surface condition	Fine and soft	4	19.05
	Moderate	16	76.19
	Coarse and hard	1	4.76
Skin thickness	Thin	4	19.05
	Moderate	16	76.19
	Thick	1	4.76
Daily UV exposure time	Less than 1 h	10	47.62
	1 to 3 h	11	52.38
	More than 3 h	0	0
Daily smoking	None	21	100
	Less than 10 pieces	0	0
	More than 10 pieces	0	0

**Table 4:** Melanin index of the control and test groups.

Group	Time	*n*	Mean	SD	Change Rate from the Baseline (%)	*p* Value vs. Baseline	*p* Value vs. Control
Control	Baseline	21	182.86	19.41	-	-	-
	2 week	21	180.68	19.39	−1.19	0.000 *	-
	4 week	21	178.57	18.39	−2.35	0.001 *	-
	6 week	21	178.90	18.71	−2.17	0.001 *	-
	8 week	21	177.38	18.04	−3.00	0.000 *	-
Test	Baseline	21	184.13	22.70	-	-	-
	2 week	21	181.40	22.17	−1.48	0.003 *	0.538
	4 week	21	178.84	22.03	−2.87	0.000 *	0.259
	6 week	21	176.92	20.67	−3.92	0.000 *	0.006 ^#^
	8 week	21	173.30	20.79	−5.88	0.000 *	0.000 ^#^

* *p* < 0.05 versus baseline of each group. ^#^ *p* < 0.05 versus control group at each time point.

**Table 5:** Erythema index of the control and test groups.

Group	Time	*n*	Mean	SD	Change Rate from the Baseline (%)	*p* Value vs. Baseline	*p* Value vs. Control
Control	Baseline	21	241.59	48.98	-	-	-
	2 week	21	230.59	48.98	−4.55	0.158	-
	4 week	21	241.14	57.20	−0.19	0.956	-
	6 week	21	240.08	50.77	−0.63	0.846	-
	8 week	21	252.75	44.09	+4.62	0.208	-
Test	Baseline	21	245.60	49.53	-	-	-
	2 week	21	230.86	41.97	−6.00	0.066	0.687
	4 week	21	250.90	49.14	+2.16	0.381	0.498
	6 week	21	245.89	38.51	+0.12	0.973	0.866
	8 week	21	252.51	39.87	+2.81	0.470	0.768

**Table 6:** Skin lightness (L* value) of the control and test groups.

Group	Time	*n*	Mean	SD	Change Rate from the Baseline (%)	*p* Value vs. Baseline	*p* Value vs. Control
Control	Baseline	21	61.28	1.77	-	-	-
	2 week	21	61.75	1.85	+0.77	0.002 *	-
	4 week	21	61.74	1.72	+0.75	0.001 *	-
	6 week	21	61.76	1.73	+0.78	0.002 *	-
	8 week	21	61.65	1.64	+0.60	0.008 *	-
Test	Baseline	21	61.33	1.82	-	-	-
	2 week	21	61.87	1.82	+0.88	0.000 *	0.469
	4 week	21	61.85	1.86	+0.85	0.000 *	0.584
	6 week	21	62.04	1.69	+1.16	0.000 *	0.009 ^#^
	8 week	21	62.19	1.48	+1.40	0.000 *	0.000 ^#^

* *p* < 0.05 versus baseline of each group. ^#^ *p* < 0.05 versus control group at each time point.

**Table 7:** Skin redness (a* value) of the control and test groups.

Group	Time Point	*n*	Mean	SD	Change Rate from the Baseline (%)	*p* Value vs. Baseline	*p* Value vs. Control
Control	Baseline	21	10.74	1.46	-	-	-
	2 week	21	10.51	1.31	−2.14	0.043 *	-
	4 week	21	10.60	1.29	−1.30	0.200	-
	6 week	21	10.71	1.47	−0.28	0.810	-
	8 week	21	10.70	1.44	−0.37	0.706	-
Test	Baseline	21	10.97	1.24	-	-	-
	2 week	21	10.62	1.07	−3.19	0.004 *	0.135
	4 week	21	10.79	1.22	−1.64	0.131	0.766
	6 week	21	10.92	1.05	−0.46	0.769	0.898
	8 week	21	10.55	1.09	−3.83	0.006 *	0.008 ^#^

* *p* < 0.05 versus baseline of each group. ^#^ *p* < 0.05 versus control group at each time point.

**Table 8:** Skin yellowness (b* value) of the control and test groups.

Group	Time	*n*	Mean	SD	Change Rate from the Baseline (%)	*p* Value vs. Baseline	*p* Value vs. Control
Control	Baseline	21	21.53	1.01	-	-	-
	2 week	21	21.58	1.03	+0.23	0.704	-
	4 week	21	21.37	1.07	−0.74	0.257	-
	6 week	21	21.35	1.02	−0.84	0.298	-
	8 week	21	21.07	1.23	−2.14	0.049 *	-
Test	Baseline	21	21.46	1.22	-	-	-
	2 week	21	21.69	1.49	+1.07	0.230	0.339
	4 week	21	21.35	1.38	−0.51	0.363	0.676
	6 week	21	21.24	1.35	−1.03	0.310	0.869
	8 week	21	21.11	1.11	−1.63	0.079	0.605

* *p* < 0.05 versus baseline of each group.

**Table 9:** Skin color (individual typology angle (ITA°)) of the control and test groups.

Group	Time	*n*	Mean	SD	Change Rate from the Baseline (%)	*p* Value vs. Baseline	*p* Value vs. Control
Control	Baseline	21	27.57	4.02	-	-	-
	2 week	21	28.49	4.21	+3.34	0.007 *	-
	4 week	21	28.73	4.05	+4.21	0.001 *	-
	6 week	21	28.76	3.69	+4.32	0.004 *	-
	8 week	21	28.92	3.92	+4.90	0.001 *	-
Test	Baseline	21	27.76	4.09	-	-	-
	2 week	21	28.67	4.31	+3.28	0.010 *	0.948
	4 week	21	28.99	4.39	+4.43	0.000 *	0.811
	6 week	21	29.56	4.24	+6.48	0.000 *	0.053
	8 week	21	30.00	3.70	+8.07	0.000 *	0.005 ^#^

* *p* < 0.05 versus baseline of each group. ^#^ *p* < 0.05 versus control group at each time point.

**Table 10:** Visually assessed degree of skin pigmentation of the control and test groups.

Group	Time	*n*	Mean	SD	Change Rate from the Baseline (%)	*p* Value vs. Baseline	*p* Value vs. Control
Control	Baseline	21	4.98	1.01	-	-	-
	2 week	21	4.98	1.01	0.00	-	-
	4 week	21	4.98	1.01	0.00	-	-
	6 week	21	4.96	1.01	−0.40	0.329	-
	8 week	21	4.92	0.99	−1.20	0.056	-
Test	Baseline	21	4.93	1.22	-	-	-
	2 week	21	4.92	1.21	−0.20	0.329	0.329
	4 week	21	4.89	1.22	−0.81	0.083	0.083
	6 week	21	4.86	1.19	−1.42	0.030 *	0.056
	8 week	21	4.76	1.15	−3.45	0.003 *	0.025 ^#^

* *p* < 0.05 versus baseline of each group. ^#^ *p* < 0.05 versus control group at each time point.

## Abbreviations

AC	adenylate cyclase
ACTH	adrenocorticotrophic hormone
CREB	cAMP-responsive element-binding protein
HPLC	high performance liquid chromatography
ITA^o^	individual topology angle
MC1R	melanocortin 1 receptor
MITF	microphthalmia-associated transcription factor
MSH	melanocyte-stimulating hormone
PKA	protein kinase A
SD	standard deviation
TYR	tyrosinase
UV	ultraviolet
